# The Effect of Bi-Functionalized MMT on Morphology, Thermal Stability, Dynamic Mechanical, and Tensile Properties of Epoxy/Organoclay Nanocomposites

**DOI:** 10.3390/polym11122012

**Published:** 2019-12-04

**Authors:** Siew Sand Chee, Mohammad Jawaid

**Affiliations:** 1 Laboratory of Biocomposite Technology, Institute of Tropical Forestry and Forest Products (INTROP), Universiti Putra Malaysia, Serdang 43400 UPM, Selangor, Malaysia; joeychee1025@gmail.com; 2 Department of Chemical Engineering, College of Engineering, King Saud University, Riyadh 145111, Saudi Arabia

**Keywords:** organoclay, montmorillonite, epoxy nanocomposites, morphology, thermal, dynamic mechanical, tensile

## Abstract

In this work, the optimum filler loading to prepare epoxy/organoclay nanocomposites by the in-situ polymerization method was studied. Bi-functionalized montmorillonite at different filler loading (0.5, 1.0, 2.0, 4.0 wt %) was dispersed in epoxy resin by using a high shear speed homogenizer. The effect on morphology, thermal, dynamic mechanical, and tensile properties of the epoxy/organoclay nanocomposites were studied in this work. Wide-angle X-ray scattering (WAXS) and field emission scanning electron microscope (FESEM) studies revealed that possible intercalated structures were obtained in epoxy/organoclay nanocomposites. Thermogravimetric analysis (TGA) shows that epoxy/organoclay nanocomposites exhibit higher thermal stability at the maximum and final decomposition temperature, as well as higher char content, compared to pristine epoxy. The dynamic mechanical analysis (DMA) indicate that storage modulus (E′), loss modulus (E″), cross-link density and glass transition temperature (*T*_g_) of the nanocomposites were improved with organoclay loading up to 1 wt %. Beyond this loading limit, the deterioration of properties was observed. A similar trend was also observed on tensile strength and modulus. We concluded from this study that organoclay loading up to 1 wt % is suitable for further study to fabricate hybrid nanocomposites for various applications.

## 1. Introduction

Epoxy resin falls under the category of thermosetting polymer which contained one or more oxirane ring structure in the molecule [1]. Epoxy resin possesses several distinct properties such as relatively high mechanical and fatigue strength, good chemical and water resistance, high adhesive strength, and excellent heat and electrical resistance. According to a report published by Market Research Future [2], the global epoxy resin market is estimated to accrue USD 10,620.5 million by 2023, posting a compound annual growth rate (CAGR) of 5.24% from 2017–2023. The positively growing market of epoxy resin is due to the increase of epoxy-based composite demand from automotive [3], electronics [4], building and construction industries [5]. Despite the excel properties of epoxy resin, their intrinsic properties such as fragility, brittleness, large thermal expansion, and low fire resistance has limited their end-use applications.

One of the approaches to overcome the shortage of pure epoxy is the incorporation of fillers. The technology advances in the field of nanotechnology and nanomaterials have brought new and interesting possibilities into the materials field with the development of polymer/clay nanocomposite where clay as a nano-sized layered silicate is dispersed in the polymer matrix. Many researchers have reported that polymer nanocomposites (PNCs) filled with small amount of nano-sized particles (< 5% by weight) show enhanced properties in several perspectives, such as improvement of mechanical properties, enhanced barrier properties, heat resistance, and fire performance [6,7]. The great breakthrough of the innovation of polymer nanocomposites was achieved by the Toyota research group when they filed their own patent for a nylon/clay nanocomposite system in 1988 [8].

Despite the success story achieved by the Toyota group, it is still challenging to obtain a fully exfoliated epoxy-clay nanocomposite which is able to meet the industrial applications requirement. The challenges lie in two main areas, which are enhancing the interactions between clay sheets by a suitable organic modifier and exfoliating the clay particles. The surface modification on nanoclay by using organic cations such as alkylammonium (R_4_N^+^X^−^) [9], alkylphosphonium (R_4_P^+^X^−^) [10] and diazonium salts (R-N^+^_2_X^−^) [11] is often used to form hydrophobic organo modified clays which can disperse more easily in polymers. Organosilane treatment is another possible way to modify the hydrophilic clay surface by forming a strong bond between the hydroxyl groups on the clay surface with the silane compound [12,13]. The first step to prepare epoxy-clay nanocomposites is to disperse the nanoclay within the epoxy resin before curing. The dispersion method is crucial to ensure the homogenous dispersion of nanoclay in the epoxy matrix which helps to facilitate the formation of the exfoliated morphology structure. The commonly used techniques for clay dispersion are mechanical stirring, ultrasonication, high shear mixing, ball milling, high pressure mixing, and the slurry process. From the literature reviewed, it shows that mechanical stirring alone is not efficient enough to prepare high performance nanocomposites [7,14,15]. High shear mixing [16] or ultrasonication [17] is often employed in the mixing process to enhance the dispersion of the nanoclay. In order to achieve the desired properties of epoxy-clay nanocomposites, it is important to understand the role of the surface modifier and how the interfacial interaction occurs. Besides that, mixing parameters such as mixing speed, mixing duration, and temperature do play an important role in the formation of the final structure/configuration (conventional, intercalated, or exfoliated) of epoxy-clay nanocomposites [18].

In recent years, utilizing a bi-functionalized nanoclay in preparation of PNCs is gaining more interest from the research community. Shakeri et al. [19] reported on a modification of organoclay with 3-trimethoxysilylpropyl methacrylate (MPS) as a reactive silane coupling agent to prepare a polymethylmethacrylate (PMMA)/OMMT nanocomposite. Their findings showed that the prepared nanocomposites increased in flexural strength, flexural modulus, and fracture toughness in comparison to the neat matrix. Yu et al. [20] carried out a study on polystyrene/OMMT nanocomposites by comparing the synergy effect of: i) phosphonium surfactant and sodium lauryl sulfate; ii) phosphonium surfactant and γ-methacryloxypropyltrimethoxysilane. The nanocomposites prepared by the cationic surfactant and silane coupling agent exhibit the best thermal stability and tribology performance. Jlassi et al. [21] reported on the modification of nanoclay with polyaniline and 4-diphenylamine diazonium forming a highly exfoliated epoxy nanocomposite.

Although bi-functionalized nanoclay seems to be more efficient, to date only limited work has been reported on the preparation of epoxy nanocomposites via this approach. Therefore, the aim of this work is to carry out studies on bi-functionalized MMT reinforced epoxy nanocomposites prepared via in-situ polymerization. The MMT inner gallery was modified by 18 carbon atoms, cationic ammonium salt (octadecylamine), and a silane coupling agent (3-aminopropyltriethoxysilane) was used to treat the surface and edges of the nanoclay to enhance dispersion in polymer resin. The dispersion method employed in this study is by mechanical stirring followed by high shear speed mixing. A set of epoxy/OMMT nanocomposites with different filler loading (0.5 wt %, 1.0 wt %, 2.0 wt % and 4.0 wt %) was prepared at room temperature curing for 24 hours. The effects of the bi-functionalized MMT on the morphological, thermal stability, dynamic, mechanical, and tensile properties of the epoxy/OMMT nanocomposites were studied. As part of our previous work [22,23,24], the data obtained from this work can be utilized to produce a high-performance natural fiber/nanoclay/epoxy hybrid nanocomposite which could potentially be used for automotive or building and construction applications.

## 2. Materials and Methods

### 2.1. Materials

Organo-modified MMT (OMMT) used in this study (Nanomer I.31PS, Nanocor) was procured from Sigma-Aldrich, Selangor, Malaysia and was used as received. The matrix used in the present study is a diglycidyl ether of bisphenol-A (DGEBA) epoxy resin with a trade name of DER-331 and a modified cycloaliphatic amine hardener with a trade name of JOINTMINE 905-3s. Both epoxy and hardener were obtained from Tazdiq Engineering Sdn. Bhd. (Selangor, Malaysia). Table 1 shows the typical properties and specifications of the OMMT, epoxy resin, and hardener.

### 2.2. Fabrication of Epoxy/OMMT Nanocomposites

**Figure 1:**
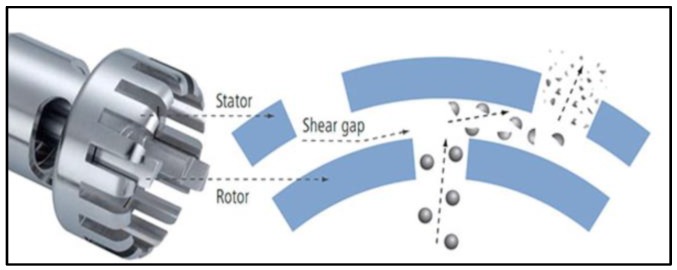
Mixing mechanism of the rotor/stator arrangement of Ultra Turrax homogenizer.

The epoxy/OMMT nanocomposites were prepared by in-situ polymerization. The OMMT at different weight fractions (0.5, 1.0, 2.0 and 4.0 wt %) were dispersed slowly into epoxy resin and mixed homogeneously by mechanical stirring at 500 rpm for 15 mins. The mixture is further mixed using a high shear speed homogenizer (T-25 Ultra Turrax Homogenizer, IKA, Staufen, Germany) with a rotation speed of 10,000 rpm for 30 min, according to a modified method reported by Al-Qadhi et al. [25]. The mixture temperature is kept at 35 °C to 45 °C with a cold-water bath to avoid excessive heat generation during the high-speed mixing. Figure 1 illustrates the mixing mechanism induced by the homogenizer. A powerful circumferential force was created by the close proximity of the outer stationary tube (stator) and the inner turning shaft (rotor) that automatically drew the nanofiller axially into the dispersion head and then forced radially through the slots in the rotor/stator arrangement. The mixture was then allowed to degas for 30 min before adding the hardener. The hardener was mixed slowly by hand stirring to avoid incurring more air bubbles. The ratio of epoxy to hardener was 2:1 according to manufacturer guidelines. Finally, the mixture was poured into a 240 mm × 120 mm × 3 mm stainless steel mold and allowed to cure at room temperature for 24 hours followed by post curing at 105 °C for 5 hours to ensure it was fully cured.

### 2.3. Characterization

#### 2.3.1. Wide-Angle X-ray Scattering

**Figure 2:**
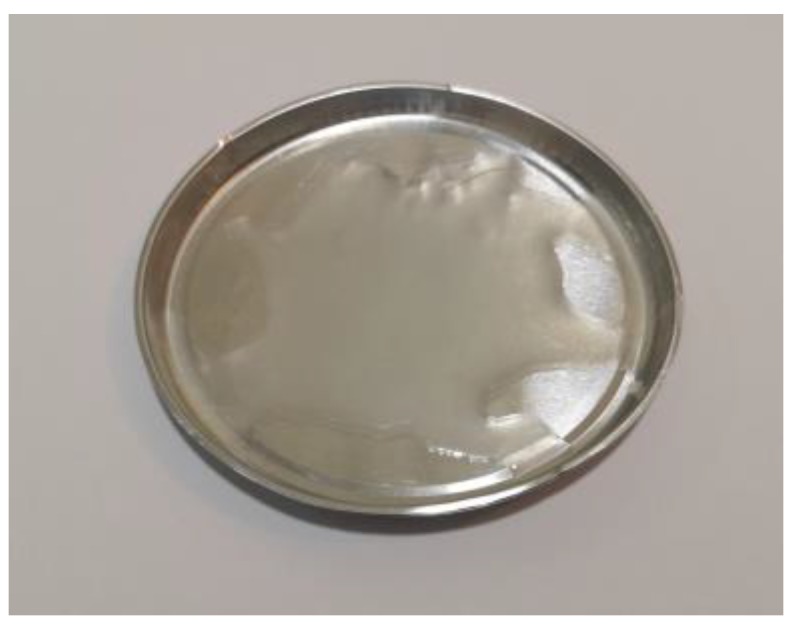
Preparation of epoxy/OMMT thin film for WAXS (Wide-angle X-ray analysis) analysis.

Wide-angle X-ray scattering (WAXS) was conducted on the OMMT powder and epoxy/OMMT nanocomposites using an X−ray diffraction unit (Model: Saxpoint 2.0, Brand: Anton Paar, Graz, Austria) with Cu-Kα radiation (wavelength = 0.1542 nm) under voltage of 50 kV and current of 1 mA. The epoxy/OMMT samples were prepared by casting a thin layer of the mixture of epoxy/nanoclay/hardener onto an aluminum pan (Figure 2) and allowing it to cure as per described earlier. The d spacing (d_001_) between the interlayer was calculated according to Bragg’s Law as per Equation (1) [26,27,28].

(1) $$d\;spacing\;\left(d001\right)=\;\frac{n\lambda }{sin\theta }$$

where, n is order 1, λ is the Cu-Kα radiation wavelength used, 2θ denotes as the peak position, which corresponds to 001 basal reflection peak of the interlayers.

#### 2.3.2. Field Emission Scanning Electron Microscope

The morphology and the dispersion state of the nanoclay in the epoxy matrix were studied using an Ultra High-Resolution Scanning Electron Microscope (FESEM) (Model: Nova NanoSem 230, Brand: FEI, Sydney, Australia). The exposure surface was sputter coated with gold to achieve better visualization.

#### 2.3.3. Thermogravimetry Analyzer (TGA)

The thermal decomposition profile of the OMMT powder and epoxy/OMMT nanocomposites were examined using a thermogravimetry analyzer (Model: TGA 1, Brand: Mettler Toledo, Greifensee, Switzerland) according to ASTM E1131-03. The samples were subjected to a temperature profile heating from 30 to 800 °C with a heating rate of 20 °C/min. All measurements were carried out under a nitrogen atmosphere with a gas flow rate of 50 mL/min.

#### 2.3.4. Dynamic Mechanical Analyzer (DMA)

The viscous elastic behavior of the nanocomposites was studied with a dynamic mechanical analyzer (Model: DMA1, Brand: Mettler Toledo, Greifensee, Switzerland) according to ASTM D5023-01. The specimens were clamped on a single cantilever measurement configuration. The specimens were pre-cut into the desired dimension with a clamping free length of 10 mm and width of 10 mm. The specimens were subjected to a heating profile scanning from 25 to 150 °C with heating rate of 5 °C/min under a frequency of 1 Hz and a displacement amplitude of 10 μm. The glass transition temperature was determined from the peak of the tan delta curve according to ASTM E1640-99.

#### 2.3.5. Tensile Testing

The tensile properties of the nanocomposites were tested by using a 10 kN capacity Universal Testing Machine model 5566 INSTRON (UTM, Instron, Norwood, MA, USA) according to ASTM D3039. The specimens were prepared in 120 mm × 20 mm × 3 mm. The gauge length used was 60 mm and a constant cross head speed of 2 mm/min was employed on the testing. The samples were placed in a preconditioning chamber for a day with a temperature of 23 ± 3 °C and relative humidity of 50% ± 10% prior to analysis. Five replicate samples were tested for each group and the average values were reported.

## 3. Results and Discussion

### 3.1. Structural and Morphological

**Figure 3:**
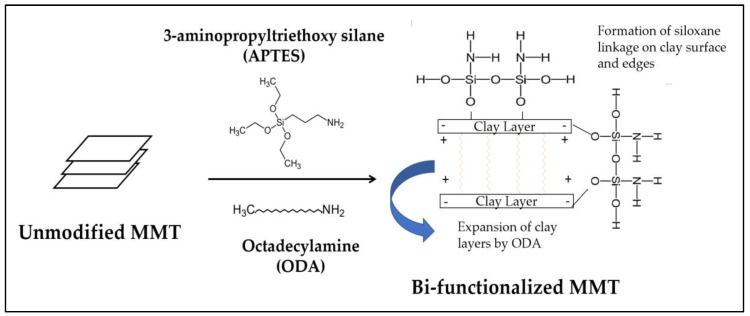
Schematic representation of the possible interaction between unmodified nanoclay, cationic ammonium salt and silane coupling agent.

Due to the fact that pristine MMTs are hydrophilic and the stacks of clay platelets are held tightly together by electrostatic forces, this led to a poor dispersion of nanoclay within the epoxy resin [7]. Surface modification is often employed to change its hydrophilicity characteristic to hydrophobicity. In this present work, a commercial bi-functionalized modified MMT was used to prepare the epoxy/OMMT nanocomposites. According to the technical datasheet, the inner galleries of MMT are treated with octadecylamine (ODA) while 3-aminopropyltriethoxy silane (APTES) is used to treat the surface and edges of the clay layers to enhance dispersion in polymer resin. Figure 3 illustrates the possible interaction between the unmodified MMT, cationic surfactant and silane coupling agent. The alkylammonium salt ions can be readily exchanged with the ions situated between the layers leading to the swelling and expansion of the interlayer space by increasing the d spacing of the clay layer [29,30]. On the other hand, a strong siloxane linkage may be formed from the reaction between the silane coupling agent with the hydroxyl groups around the surface or edges of the clay layers [31].

**Figure 4:**
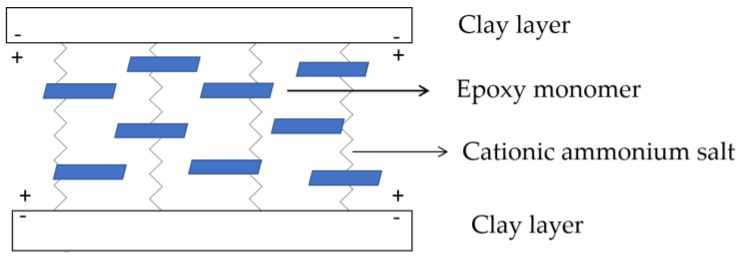
Schematic presentation on the diffusion of epoxy monomer into the clay galleries.

**Figure 5:**
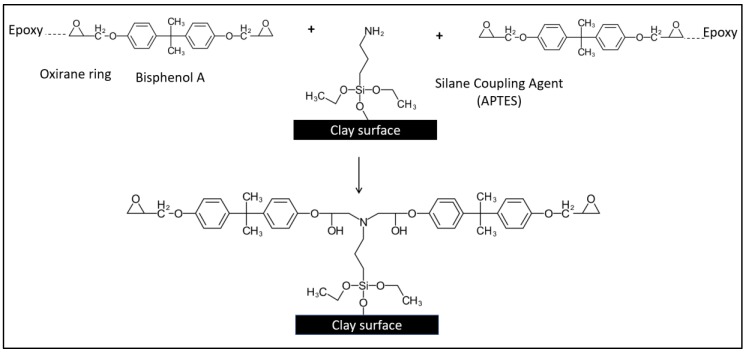
Chemical reaction between clay layer, silane coupling agent and epoxy monomer.

In this work, epoxy/OMMT nanocomposites containing 0.5, 1.0, 2.0 and 4 wt % nanoclay were prepared by using a high shear speed homogenizer. The morphology and state of dispersion of the nanoclay in epoxy resin were investigated using WAXS and the findings were further confirmed with FESEM. The positively charged ammonium salt is attracted to the negatively charged clay sheets and the long carbon chains of the ammonium salt will extent away from the stacking clay layers. As a result of this, the electrostatic force between the silicate layers were reduced, thus facilitating the diffusion of the epoxy monomer into the clay galleries which induces intercalated or exfoliated morphology (Figure 4) [14]. Besides that, the interfacial adhesion between epoxy and the clay layer is improved due to the formation of strong siloxane linkage around the surface and edges of clay layers. The amine group (–NH_2_) of APTES can react with the oxirane ring of the monomer of DGEBA (Figure 5).

**Figure 6:**
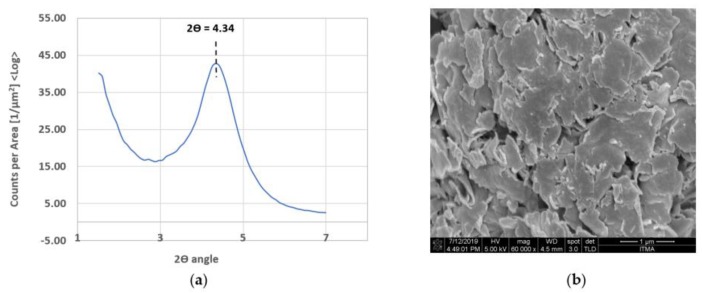
(**a**) WAXS pattern of OMMT powder; (**b**) FESEM image of OMMT powder at 60 × 10^3^ magnification.

Figure 6a,b shows the WAXS pattern and FESEM image of the OMMT powder. From the WAXS, it shows a single sharp peak at 4.34° of 2θ value, which corresponds to the 001 basal reflection peak of the interlayers. The interlayer distance of the OMMT powders calculated from Equation (1) is 2.038 nm and this matches with the technical specification in Table 1. From the FESEM image, OMMT powder appears to be a soft plate like structure, consisting of two tetrahedral silica layers sandwiching an octahedral alumina layer.

**Figure 7:**
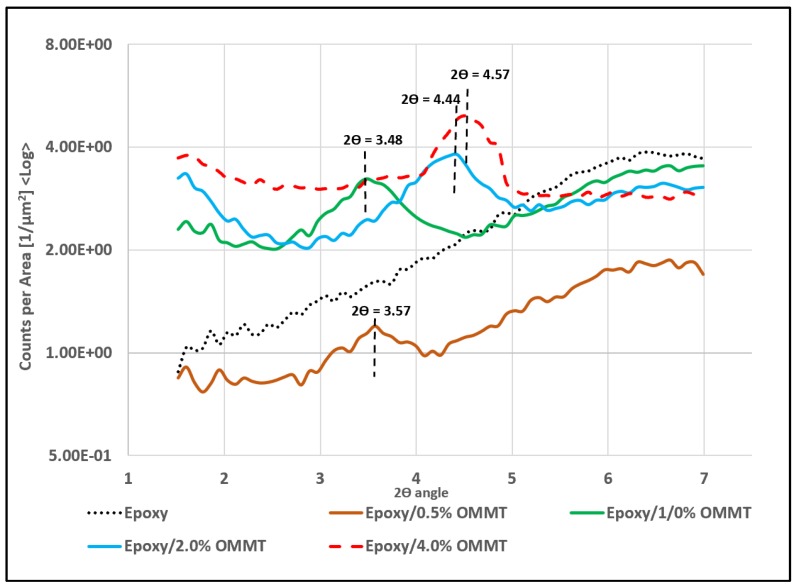
WAXS pattern of epoxy/OMMT nanocomposites with different concentration of OMMT.

The WAXS pattern of the epoxy/OMMT nanocomposite with a different concentration of OMMT is shown in Figure 7. The WAXS for pristine epoxy was also displayed for comparative purposes. In the range of 3° to 5° of 2θ value, the author observed peaks for all types of epoxy/OMMT nanocomposites. However, no peak was observed on pure epoxy, suggesting the existence of the OMMT in the epoxy matrix. The 2θ peak of all nanocomposites and its corresponding d spacing are tabulated in Table 2.

**Figure 8:**
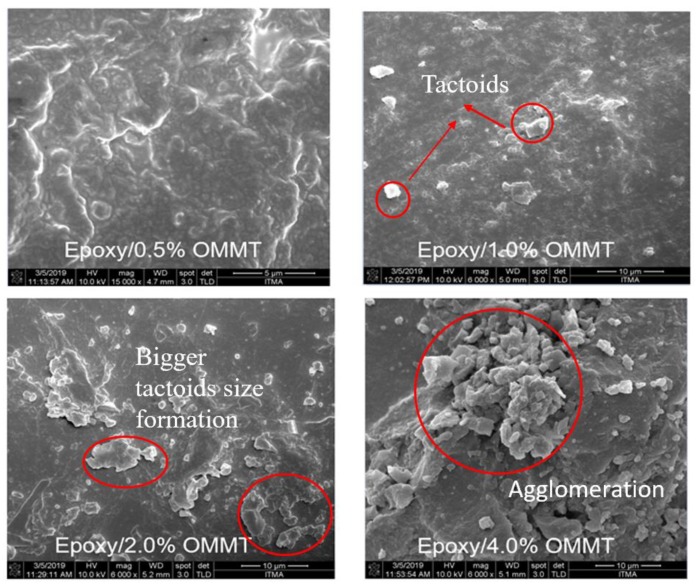
FESEM images of epoxy/OMMT nanocomposites.

The author observed that the characteristic peak of OMMT in the prepared nanocomposites had shifted to a lower angle for nanocomposites with 0.5 wt % and 1.0 wt % OMMT. The d spacing increases from 2.04 nm to 2.48 nm and 2.54 nm, respectively. The increment of d spacing indicates that there is good interaction between epoxy and OMMT, suggesting intercalated nanocomposites have been formed. A similar observation has been reported by [32,33]. In contrast, nanocomposites with 2 wt % and 4 wt % of OMMT, the 2θ peak was found to shift to higher with decreasing d spacing compared to OMMT. This may be attributed to the high loading of nanoclay leading to poor dispersion. Eventually agglomeration and stacking between the clay layer has occurred, resulting in smaller d spacing. The dispersion state of the nanoclay within the epoxy matrix were further confirmed with FESEM images (Figure 8). The images confirmed that the nanoclay in epoxy/0.5% OMMT and epoxy/1.0% OMMT nanocomposites exhibit a more well dispersed state where less tactoids formation was observed. With a higher concentration of OMMT, bigger tactoids size and more agglomeration was observed in epoxy/2% OMMT and epoxy/4% OMMT nanocomposites, this correlates well with the findings from WAXS. From the structural and morphological study, epoxy/1% OMMT nanocomposite shows the best nanoclay dispersion state with the highest d spacing recorded among all the prepared nanocomposites, suggesting the formation of intercalated nanocomposites.

**Figure 9:**
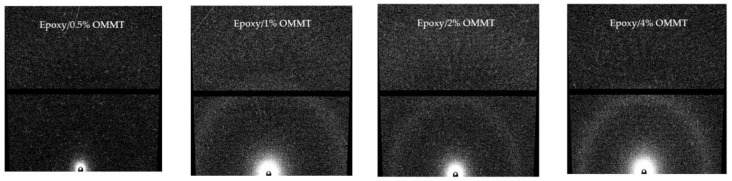
2D SAXS signal of epoxy/OMMT nanocomposites.

Figure 9 presented the 2D SAXS (small angle X-ray scattering) signal of epoxy/OMMT nanocomposites. The 2D plots provides insight about the polymer structure. The isotropic sample typically displays a complete ring, while the oriented sample often displays two or more bright maxima [34,35]. The 2D SAXS signal of all epoxy/OMMT nanocomposites shows a ring pattern, indicating the samples are isotropic with no preffered orientation of the nanostructure within the plane of the samples.

### 3.2. Thermal Stability Properties

**Figure 10:**
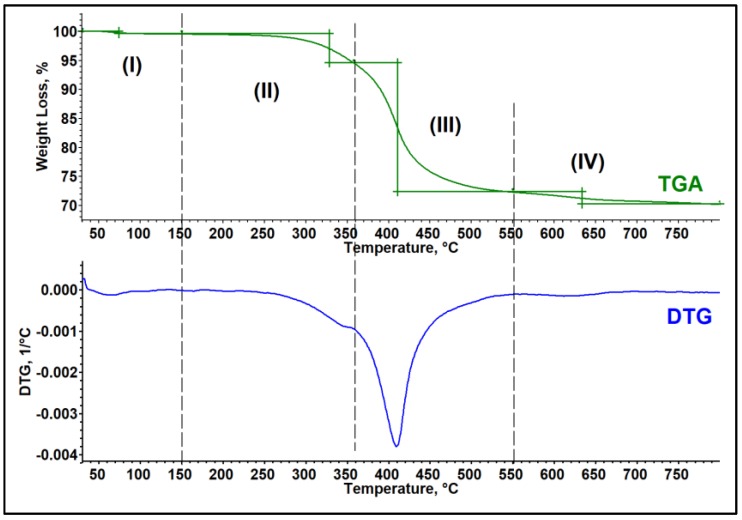
TGA, DTG curves and its corresponding weight loss steps of OMMT powder.

Thermal stability of the OMMT powder and epoxy/OMMT nanocomposites were accessed by TGA under nitrogen atmosphere. The curves and data evaluation were done by METTLER TOLEDO STARe software (Mettler Toledo, Greifensee, Switzerland). The TGA and DTG (derivative thermo gravimetric) curves of OMMT powder are shown in Figure 10. The details of the corresponding weight loss step are tabulated in Table 3. According to the DTG curve of OMMT, the decomposition profile of OMMT reveals four weight loss steps. The first weight loss step takes place in the temperature region of 30–150 °C with weight loss of 0.41%, which can be related to the moisture evaporation. From the TGA curve, the major decomposition was observed to happen in the temperature range of 200–550 °C. However, from the DTG curve it reveals there is a relatively small weight loss step observed in the temperature range of 150–358 °C with weight loss of 5% (weight loss step (II)), followed by the major weight loss in the temperature range of 358–550 °C with weight loss of 22% (weight loss step (III)). The second weight loss step can be attributed to the decomposition of APTES and third weight loss step attributed to the decomposition of ODA [36]. According to data published by the PubChem-National Center for Biotechnology Information, the decomposition temperature for APTES is around 217 °C [37] while ODA decomposes at around 350 °C [38]. In addition, the percentage of weight loss also correlates well with the composition of the surface modifier as per technical specification in Table 1. At higher temperatures of around 650 °C, another small weight loss of 2.12% was observed which can correlate to the dehydroxylation of the clay layers [20,39]. The reaction process may be shown chemically as below [40]:

$${\mathrm{OH}}^{-}\;⟷\;H^{+}+{\;O}^{2-}{\;\mathrm{and}\;H}^{+}+{\;\mathrm{OH}}^{-}\;⟷{\;H}_{2}O$$

**Figure 11:**
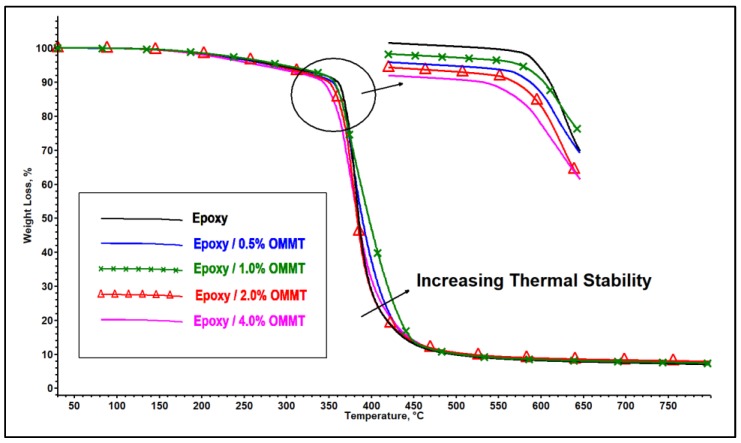
TGA curves of epoxy and epoxy/OMMT nanocomposites.

The thermal decomposition profile of epoxy nanocomposites with 0.5, 1.0, 2 and 4 wt % of OMMT is shown in Figure 11. The thermal decomposition profile of pristine epoxy also is shown for comparison purposes. The initial decomposition (*T*_IDT_), maximum decomposition (*T*_MAX_), and final decomposition (*T*_FDT_) temperature of the composites are summarized in Table 4. The initial decomposition temperature of the nanocomposites was found lower compared to pristine epoxy. The *T*_IDT_ of epoxy is 365 °C, while the *T*_IDT_ for nanocomposites with 0.5, 1, 2 and 4 wt % of OMMT are 353, 356, 347 and 336 °C, respectively. The reduction in thermal stability at a low temperature may be due to the existence of the organic surface modifier on clay which exhibits lower thermal stability. In addition, the ammonium salt that attached to the clay may act as a catalyzer towards the degradation of the polymer matrix through the Hoffman degradation mechanism; a similar observation has been reported by several researches [33,41,42,43]. The *T*_IDT_ of the epoxy nanocomposites decreases as the concentration of the OMMT increases. This may be due to the increased amount of surface modifier in the epoxy matrix leading to the acceleration of the decomposition reaction of the polymer matrix.

The presence of the nanoclay has improved the thermal stability of the epoxy nanocomposites at a higher temperature range compared to pristine epoxy as illustrated in Figure 11. The optimum performance was observed on epoxy/1% OMMT nanocomposite with the highest *T*_MAX_ and *T*_FDT_ among all the composites. With increasing OMMT concentration, the *T*_MAX_ and *T*_FDT_ of the corresponding nanocomposites decrease, yet it is still higher compared to pristine epoxy. In addition, the char formation of epoxy nanocomposites was also found to be higher compared to pristine epoxy. The decomposition temperature at different weight loss percentages of 10%, 30%, 50%, 80% and 90% and char residue at 800 °C are reported in Table 5. The improvement of the thermal stability at a high temperature may be attributed to the silicate layer acting as a protective barrier to hinder the moving out of volatile products generated during the decomposition process. In addition, the charring capability induced by clay minerals through its catalytic effects and reinforcement of char structure is also an important characteristic to improve the thermal stability of the nanocomposites by reducing the mass transport and permeability of oxygen [44,45]. Thus, epoxy/1% OMMT nanocomposites reveal the best thermal stability performance due the best intercalation morphology obtained among all the nanocomposites. As filler loading increases, this induces more agglomeration and reduces the effectiveness of forming intercalation morphology composites. This may lead to reduction of char building capability which cannot effectively protect the polymer matrix. This can be proven by the char residue for 2% and 4% OMMT nanocomposites showing relatively low increments in char residue despite the high loading of OMMT.

### 3.3. Dynamic Mechanical Properties

**Figure 12:**
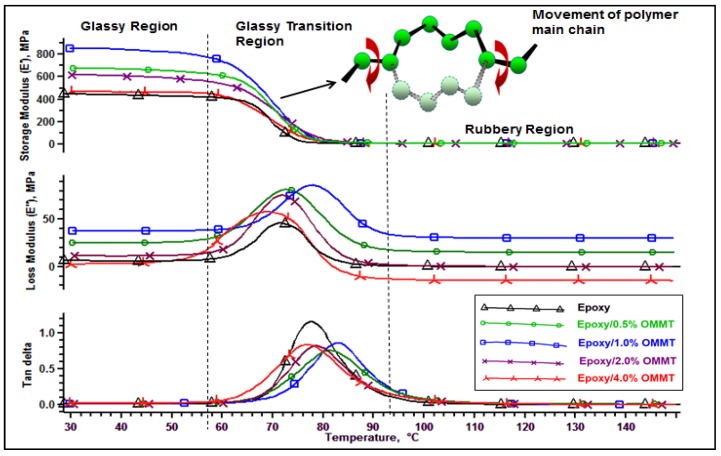
Storage modulus (**upper**), loss modulus (**middle**) and tan delta (**bottom)** curves of epoxy and epoxy nanocomposites as a function of temperature.

The effect of the organoclay loading on the viscoelastic behavior of the nanocomposites as a function of temperature and frequency under an oscillating force was studied by the DMA. The storage modulus (E′), loss modulus (E″), and tan delta curves of the pristine epoxy and epoxy nanocomposites are shown in Figure 12, and the obtained DMA parameters data are summarized in Table 6 and Table 7. The ability of the material to store or return energy is represented by E′ and provides valuable insight on the load bearing capacity [46], stiffness [23], degree of crosslinking [47], and fiber/matrix interfacial bonding [48]. The E′ decreases as the temperature increases with a steep change in modulus was observed in the temperature range of 60–90 °C. This can be denoted as the glass transition (*T_g_*) region or α relaxation of the polymer whereby it represents the movement of the polymer main chain. Below the glass transition region, the movement of the polymer chain was restricted due to the low mobility of the frozen and closely packed molecules arrangement. As a result, the E′ is high in the glassy state. With temperature increases, the closely packed molecule arrangement collapses resulting in high molecular mobility and increases the free volume components, thus resulting in a drastic fall of modulus and moving into the rubbery region of the material.

An increasing loading of OMMT increases the E′ in both glassy and rubbery regions as shown in Table 6. The storage modulus in the glassy state is represented by E′ at 25 °C while the storage modulus in rubbery region is represented by È at (*T*_g_ + 30) °C. With 0.5 and 1.0 wt % of OMMT loading, the E′ at the glassy region improved by 50% and 87% respectively compared to pristine epoxy. With filler loading at 2 wt %, we observed a decrease in modulus with E′ recorded at 615MPa and a further reduction in E′ was observed on epoxy/4% OMMT nanocomposites with the recorded E′ of 466 MPa. A similar trend was also observed on the storage modulus in the rubbery region. The improvement of the E′ in both glassy and rubbery region can be attributed to the incorporation of the OMMT limiting the movement of the epoxy polymer chain as they intercalated in between the clay layers of OMMT [49]. The decrease in modulus at a higher clay content (≥2 wt % ) is due to the poorly dispersed nanoclay in the epoxy resin with the aggregation of particles [50]. On the other hand, the cross link density can be calculated from the kinetic theory of rubber elasticity from the DMA data according to Equation (2) [47].

(2) $$\nu_{e}=\frac{È}{3RT}$$

where $\nu_{e}$ is the crosslink density (mol/m^3^), È is the storage modulus at *T* = (*T*_g_ + 30) °C, R is the gas constant (8.3145 J/K mol) and T is temperature in Kelvin. It is evident that the increasing loading of the OMMT improved the crosslink density compared to pristine epoxy. The crosslink density of epoxy raised from 6.1 × 10^−4^ to 10.2 × 10^−4^ (mol/m^3^) (Table 6). The obtained results are in line with other research findings for functionalized soybean oil modified toughened epoxy/organoclay [51]. As discussed earlier, silane coupling agent induced better interfacial bonding between organoclay and epoxy matrix thus resulting in a high crosslink density. As a result, the flexibility of the material is being reduced due to the segmental mobility of the polymer molecules being constrained. However, a decrease in crosslink density is observed on Epoxy/2% OMMT and Epoxy/4% OMMT. This can be attributed to the nanoclay agglomeration which affected the curing reaction and reduces when the cross link occurs. A similar argument is also reported by [52].

The obtained loss modulus (E″) was summarized in Table 7. E″ describes the viscous response of a material where the energy dissipated during the stress cycle was measured. In the glass transition region, the E″ exhibits a distinct peak indicating high losses of energy caused by the internal friction and non-elastic deformation in the molecular segmental motion. Improvement of loss modulus was also observed from the plot of loss modulus vs. temperature as the filler loading of OMMT increases. The loss modulus curve for epoxy nanocomposites appears to be broader and increased in peak height compared to pristine epoxy. A similar observation also reported by other researchers is that the incorporation of nanoclay induces internal friction and leads to high energy dissipation [49,53]. Epoxy/1% OMMT nanocomposites marks the highest peak of loss modulus followed by epoxy/0.5% OMMT and epoxy/2.0% OMMT nanocomposite. Epoxy/4% OMMT nanocomposite exhibits the lowest peak of E″ among all epoxy nanocomposites. Besides that, all epoxy nanocomposites of E″ display a broader peak shape compared to pristine epoxy which indicates the free volume and chain segments of the polymer matrix were increased with the addition of nanoclay.

Lastly, the loss factor or Tan delta curve corresponds to the ratio of loss modulus to storage modulus and its maximum peak height can be denoted as the glass transition temperature (*T*_g_) of the material. Table 7 summarized the peak height of tan delta and *T*_g_ by the peak of tan delta. The tan delta curve of pristine epoxy appears to be a sharp and narrow peak with the highest tan delta value recorded at 1.15 with *T*_g_ recorded at 77.8 °C. A high tan delta value indicates high damping behavior or a high degree of non-elastic deformation. On the contrary, a low value indicates that the material is more elastic. With the incorporation of organoclay, the tan delta curve of epoxy nanocomposites appears to be broader and decreases in peak intensity with the *T*_g_ also found to be shifted to a higher temperature. The lower tan delta value and broad peak is evidence that the improving damping behavior is due to the presence of the intercalated layers of OMMT modifying the network structure, effecting the relaxation mechanisms and arresting the segmental motion near the organic-inorganic interface. These findings are in line with other research findings [50,51,54,55]. Additionally, the author observed that increasing crosslink density shifts the *T*_g_ higher. The increase of *T*_g_ can be attributed to the constraint of relaxation mobility in the polymer segments caused by the chemical bonding around the interface of the clay layer and epoxy matrix [55]. Epoxy/1% OMMT nanocomposite reveal the highest crosslink density (10.2 × 10^−4^ mol/m^3^) with the highest *T*_g_ (82.6 °C) among all composites. This is followed by the Epoxy/0.5% OMMT nanocomposite with a crosslink density of 9.2 × 10^−4^ mol/m^3^ and its *T*_g_ recorded at 81 °C. However, lacking surrounding entanglements and reduced crosslink density at the interface decreases the *T*_g_. This trend was observed on epoxy nanocomposites with 2 and 4 wt % of OMMT loading.

### 3.4. Tensile Properties

**Figure 13:**
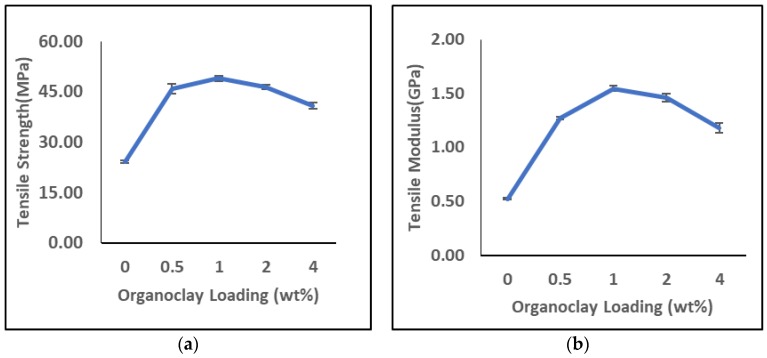
Effect of organoclay loading on: (**a**) tensile strength; (**b**) tensile Modulus.

The tensile properties of epoxy organoclay nanocomposites strongly depends on the structure or configuration of nanocomposites, i.e., conventional/intercalated/exfoliated and interfacial interaction between polymer and clay layers [56]. The tensile properties of the pristine epoxy and the nanocomposites on the effect of different OMMT loading are shown in Figure 13. From the analysis, it was observed that the addition of OMMT improved the tensile strength and modulus with increasing the clay loading up to 1 wt %. With 0.5 wt % and 1.0 wt % of OMMT have improved the tensile strength by 90% and 103%, respectively, while the tensile modulus has improved by 143% and 200%, respectively. The reinforcing effect of the clay layers on the tensile properties is mainly due to the inherently high moduli and high aspect ratio of the clay particles [14,55]. The high aspect ratio of organoclay induces more surface contact area with polymer matrix and it can act efficiently as a stress transfer agent in the composites, thus improving the tensile properties. This improvement is also evidence that the nanoscale dispersion is prevalent with the formation of the intercalated structure with the bi-functionalized organoclay through the ultra-high shear speed mixing. It has been reported extensively that surface modification on clay results in better polymer-clay interfacial adhesion with improvement of tensile properties (Table 8).

**Figure 14:**
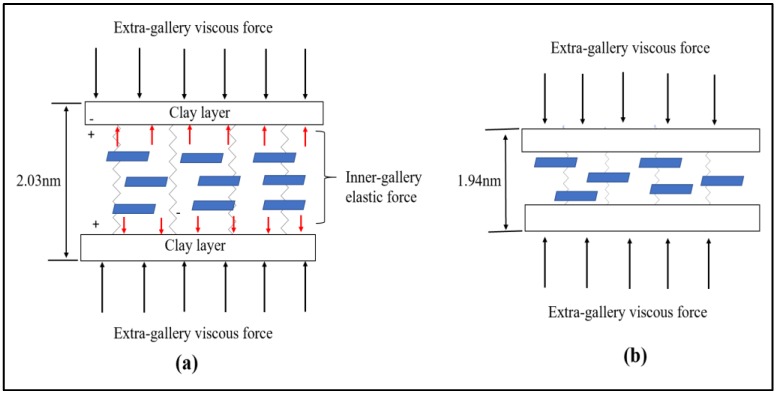
Schematic illustration on the forces acting on a pair of clay layer: (**a**) intercalated/exfoliated state (**b**) tactoids formation.

Beyond 1 wt % of OMMT loading, the tensile properties of the nanocomposites start to decline. A similar phenomena is also reported elsewhere [32,45,58]. Several possible reasons may contribute to this observation, such as the micro voids formation during sample preparation due to the weak boundaries between the particles and air-trapped bubbles [59]. Another possible reason is the organoclay induces epoxy self-polymerization which increases the viscosity of epoxy/organoclay mixture as organoclay loading increases [60]. The amine base organic modifier used in this work may partially contribute to curing during the mixing process of epoxy resin and organoclay which could lead to heterogeneity in the resultant samples [55]. During the preparation of the nanocomposites, we do observe the viscosity of the epoxy/organoclay mixture become more viscous with higher loading of organoclay. Due to the high viscosity of the mixture, it is more difficult to cast the mixture into mould. This eventually lead to more air-trapped bubbles and void formation which can act as the weak point of the samples. As the curing is being accelerated by the organic surface modifier, the extra-gallery viscous force outweighs the inter-gallery elastic force between the clay layers thus leading to tactoids formation (Figure 14) [61]. This can be proven from the d spacing obtained from the WAXS study, the d spacing for epoxy/2% OMMT and epoxy 4%/OMMT nanocomposite reduces by 2.25 % (1.992 nm) and 5.05% (1.935 nm) respectively compared to pristine epoxy (2.038 nm). Moreover, tactoids formation leading to the agglomeration of nanoclay could give a stress concentration effect and reduce the tensile strength of nanocomposites.

## 4. Conclusions

Epoxy/organoclay nanocomposites with a different organoclay concentration were prepared by high shear speed homogenizer through in-situ polymerization in the presence of bi-functionalized MMT. The WAXS and FESEM suggested that intercalated epoxy/organoclay nanocomposites were obtained on epoxy/0.5% OMMT and epoxy/1.0% OMMT nanocomposites. The FESEM images confirmed that nanocomposites with OMMT loading beyond 1 wt % exhibits large tactoids particles and agglomeration. The TGA analysis shows that the thermal stability of the nanocomposites was improved at a higher temperature range. The initial decomposition of all nanocomposites was lower compared to pristine epoxy due to the existence of the organic surface modifier which possesses lower thermal stability. The E′, and E″ of the nanocomposites were greatly improved with the incorporation of organoclay up to 1 wt % loading of OMMT. The DMA results also reveal that epoxy/1% OMMT nanocomposites exhibit the highest crosslink density and *T*_g_ value among all the composites. A similar observation is also observed on the tensile properties of the nanocomposites with the highest tensile strength and modulus was observed on epoxy/1% OMMT nanocomposite. Overall, it can be concluded that 1.0 wt % OMMT filler loading epoxy nanocomposites shows considerable higher thermal stability, viscous elastic properties, crosslink density, *T*_g,_ and tensile properties.

## Figures and Tables

**Table 1:** Typical properties and specification of OMMT (Organo-modified MMT), epoxy resin, and hardener.

**OMMT**	**Value**
Surface Modifier (wt %)	
Octadecylamine	15–35
3-aminopropyltriethoxy silane	0.5–5
Mean Particle size (Micron)	14–18
d spacing (d_001_) (nm)	1.8–2.2
**Epoxy**	**Value**
Epoxy equivalent weight (g/eq)	182–192
Epoxide percentage (%)	22.4–23.6
Viscosity at 25 °C (mPa.s)	11,000–14,000
Density at 25 °C (g/ml)	1.16
**Hardener**	**Value**
Amine value (mg KOH/g)	300 ± 20
Viscosity (BH type at 25 °C, cPs)	200–400

**Table 2:** Peak in 2θ of WAXS curve and its corresponding d spacing of OMMT powder and epoxy/OMMT nanocomposites.

Specimens	Peak in 2*θ* (°)	d Spacing (nm)
OMMT	4.34	2.038
Epoxy/0.5% OMMT	3.57	2.476
Epoxy/1.0% OMMT	3.48	2.54
Epoxy/2.0% OMMT	4.44	1.992
Epoxy/4.0% OMMT	4.57	1.935

**Table 3:** Decomposition profile of OMMT powder measured with TGA under nitrogen atmosphere.

Weight Loss Step	Temperature Range (°C)	Weight Loss (%)	Possible Reaction
I	30–150	0.41	Moisture evaporation
II	150–358	5	Decomposition of APTES
III	359–550	22.3	Decomposition of Octadecylamine
IV	550–800	2.12	Dehydroxylation of clay layers

**Table 4:** Initial decomposition, maximum decomposition and final decomposition temperature of the epoxy nanocomposites.

Composites	*T*_IDT_/°C	*T*_MAX_/°C	*T*_FDT_/°C
Epoxy	365	383	425
Epoxy/0.5% OMMT	353	387	449
Epoxy/1% OMMT	356	394	461
Epoxy/2% OMMT	347	381	446
Epoxy/4% OMMT	336	381	458

***T*_IDT_**: Initial decomposition temperature; ***T*_MAX_**: Maximum decomposition temperature; ***T*_FDT_**: Final decomposition temperature.

**Table 5:** Decomposition temperature at total mass loss of 10%, 30%, 50%, 80%, 90% and residue at 800 °C.

Composites	*T*_10%_ (°C)	*T*_30%_ (°C)	*T*_50%_ (°C)	*T*_80%_ (°C)	*T*_90%_ (°C)	Residue at 800 °C (%)
Epoxy	361	377	385	418	494	5.98
Epoxy/0.5% OMMT	355	377	386	423	513	6.58
Epoxy/1% OMMT	357	378	397	434	527	7.27
Epoxy/2% OMMT	350	373	383	419	499	7.67
Epoxy/4% OMMT	342	369	384	425	503	7.76

**Table 6:** Storage modulus at glassy and rubbery state and cross link density of epoxy and epoxy nanocomposites.

Composites	Storage Modulus (E’) at 25 °C (MPa)	Storage Modulus (È) at *T*_g_ + 30 °C (MPa)	Crosslink Density $\left(\nu_{e}\right)$ (×10^−4^ mol/m^3^)
Epoxy	449	6	6.1
Epoxy/0.5% OMMT	675	9	9.2
Epoxy/1.0% OMMT	850	10	10.2
Epoxy/2.0% OMMT	615	8.4	8.6
Epoxy/4.0% OMMT	466	7.8	8

**Table 7:** Summary of DMA parameters of epoxy and epoxy nanocomposites.

Composites	Peak of Loss Modulus (MPa)	Peak of Loss Modulus (°C)	Peak of Tan Delta	*T*_g_ by peak of Tan Delta (°C)
Epoxy	45.8	71.6	1.16	77.8
Epoxy/0.5% OMMT	81.5	73	0.76	81
Epoxy/1.0% OMMT	85.8	77.9	0.86	82.6
Epoxy/2.0% OMMT	75.4	72	0.82	78.4
Epoxy/4.0% OMMT	57.8	69.4	0.84	76.6

**Table 8:** Reported work on improvement of tensile properties with surface modified MMT/epoxy system.

Type of Modifier	Method of Dispersion	Tensile Strength (%)	Tensile Modulus (%)	Reference
Polyaniline, DPA ^a^	Sonication	+68	-	[21]
Octadecylammonium	High shear mixing	+7	+42	[16]
3-aminopropyl-trimethoxysilane	Slurry mixing	+25	+13	[57]

^a^ 4-diphenylamine diazonium.
